# Plasma miR-324-3p and miR-1285 as diagnostic and prognostic biomarkers for early stage lung squamous cell carcinoma

**DOI:** 10.18632/oncotarget.11198

**Published:** 2016-08-11

**Authors:** Xujie Gao, Yang Wang, Hua Zhao, Feng Wei, Xinwei Zhang, Yanjun Su, Changli Wang, Hui Li, Xiubao Ren

**Affiliations:** ^1^ Department of Immunology, Tianjin Medical University Cancer Institute and Hospital, Tianjin, China; ^2^ Department of Biotherapy, Tianjin Medical University Cancer Institute and Hospital, Tianjin, China; ^3^ National Clinical Research Center of Cancer, Tianjin, China; ^4^ Key Laboratory of Cancer Immunology and Biotherapy, Tianjin, China; ^5^ Department of Lung Cancer, Tianjin Medical University Cancer Institute and Hospital, Tianjin, China

**Keywords:** lung squamous cell carcinoma, plasma, miRNA, diagnostic marker, prognostic marker

## Abstract

**Background:**

Specific biomarkers for early detection and outcome prediction of lung squamous cell carcinoma (LSCC) are still lacking. This study assessed the differentially expressed miRNAs as potential biomarkers for early stage LSCC.

**Results:**

Base on the results of multi-phase study, we found that miR-324-3p was significantly up-regulated, whereas mir-1285 was significantly down-regulated in plasma of stage I LSCC patients compared to healthy controls. ROC analysis showed that AUC of miR-324-3p and miR-1285 were 0.79 and 0.85, respectively. The combination of these two miRNAs could further improve the diagnostic accuracy (AUC = 0.89). The multivariate analysis revealed that plasma miR-324-3p level was an independent prognostic predictor for early stage LSCC.

**Methods:**

395 patients and 195 healthy controls were enrolled in this study. We screened the differentially expressed plasma miRNAs using TaqMan Low Density Arrays (TLDA) followed by three-phase qRT-PCR validation. We also evaluated the association of candidate miRNAs with overall survival of early stage LSCC patients. Finally, the target genes of the candidate miRNAs were analyzed using public available databases and bioinformatics methods.

**Conclusions:**

The current study suggests that plasma miR-324-3p and miR-1285 levels could serve as LSCC early detection markers while miR-324-3p may serve as a prognostic marker for LSCC patients.

## INTRODUCTION

Lung cancer is one of the most common diagnosed malignancies and the leading cause of cancer mortality globally [1]. Despite the remarkable improvements in detection, prevention, and treatment options (surgery and chemoradiation and targeting therapy), the survival of advanced stage patients remains poor. However, the 5-year survival rate of non-small cell lung cancer (NSCLC) patients diagnosed at an early stage could reach 80% [2]. Unfortunately, effective biomarkers for early diagnosis and prediction of prognosis for lung cancer patient are still lacking. Although the National Lung Screening Trial (NSLT) reported a 20% reduction in mortality of high-risk individuals with annual chest radiography using low-dose computed tomography (LDCT) screening, the high false-positive rates and the potential hazard of LDCT screening may limit its application [3]. Therefore, there is an urgent need to identify a non-invasive biomarker for early detection and outcome prediction in lung cancer.

MiRNAs (miRNAs) are small, non-coding RNAs and function as regulators of gene expression at posttranscriptional level [4]. By binding to the 3′- or 5′-untranslated region (UTR) of the target genes [5, 6], miRNAs are able to suppress mRNA-protein translation or to promote mRNA degradation; thus, miRNAs play an important role in cell proliferation, differentiation, and apoptosis and therefore to regulate embryo development and homeostasis [4–6]. Aberrant expression of miRNAs was observed in various human cancers. Depending on the target genes, miRNAs can function as either tumor suppressors or oncogenes by regulating genes that are associated with tumorigenesis. Moreover, miRNAs can be released into the circulation and stay there in a remarkably stable form. This suggested that circulating miRNAs could serve as noninvasive biomarkers for cancer diagnosis and prognosis [7].

Lung squamous cell carcinoma (LSCC) is the second most common type of NSCLC. The genomic and epigenomic landscape between LSCC and lung adenocarcinoma (LAD) are quite different [8]. Although a number of studies have identified specific circulating miRNAs as potential diagnostic and prognostic markers for NSCLC, most of these studies involved advanced stage patients and did not investigate the difference of miRNAs expression pattern between LSCC and LAD. Thus, in this study, we aim to assess the global plasma miRNA expression profile of patients with stage I LSCC in order to identify miRNAs that are able to serve as markers for early detection of LSCC. Furthermore, the role of selected miRNAs in the prognosis of LSCC patients will be assessed.

## RESULTS

### Characteristics of patients

**Figure 1:**
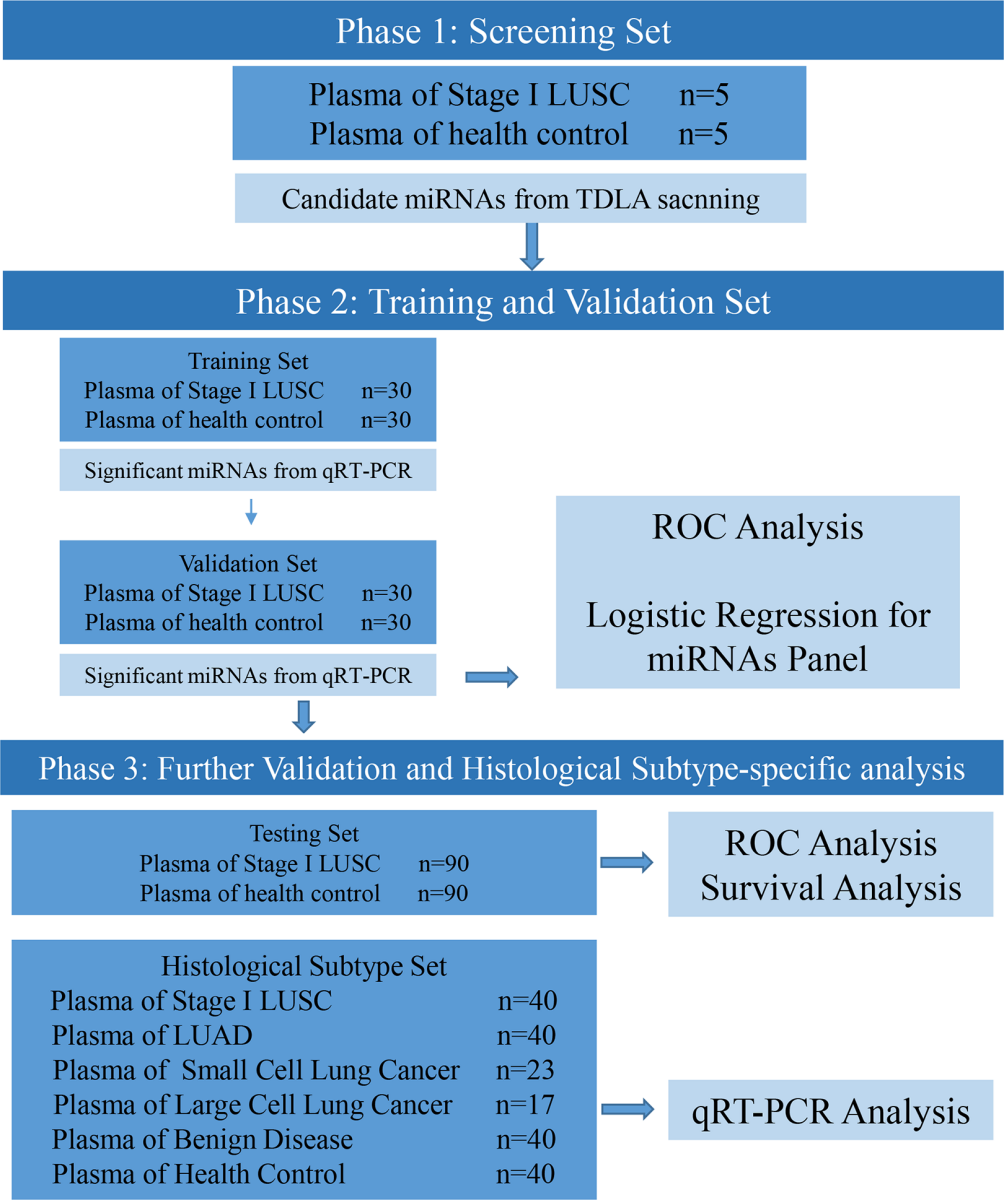
Overview of the study design

The characteristics of enrolled patients and cancer-free controls are summarized in Table 1. No significant difference was observed in age and gender between patients and controls. The overview of the study is illustrated in Figure 1.

**Table 1:** Characteristics of the study population

Phase	variable	case	control	*P*
Training set	Mean age, years ± SD	61.07 ± 9.15	60.23 ± 9.09	0.725^*^
(case = 30, control = 30)	Sex			
	Male	28 (82.9%)	26 (84.0%)	0.671^†^
	Female	2 (17.1%)	4 (16.0%)	
Validation set	Mean age, years ± SD	63.20 ± 6.19	62.33 ± 6.48	0.598^*^
(case = 30, control = 30)	Sex			
	Male	28 (93.3%)	27 (90/0%)	1.000^†^
	Female	2 (6.7%)	3 (10.0%)	
Testing set				
(case = 90, control = 90)	Mean age, years ± SD	61.78 ± 9.21	62.37 ± 8.77	0.651^*^
	Sex			
	Male	72 (80.0%)	70 (77.8%)	0.855^†^
	Female	18 (20.0%)	20 (22.2%)	

* *P* for student *t* tests

† *P* for χ^2^ tests.

### MiRNA expression profiling by TLDA

**Figure 2:**
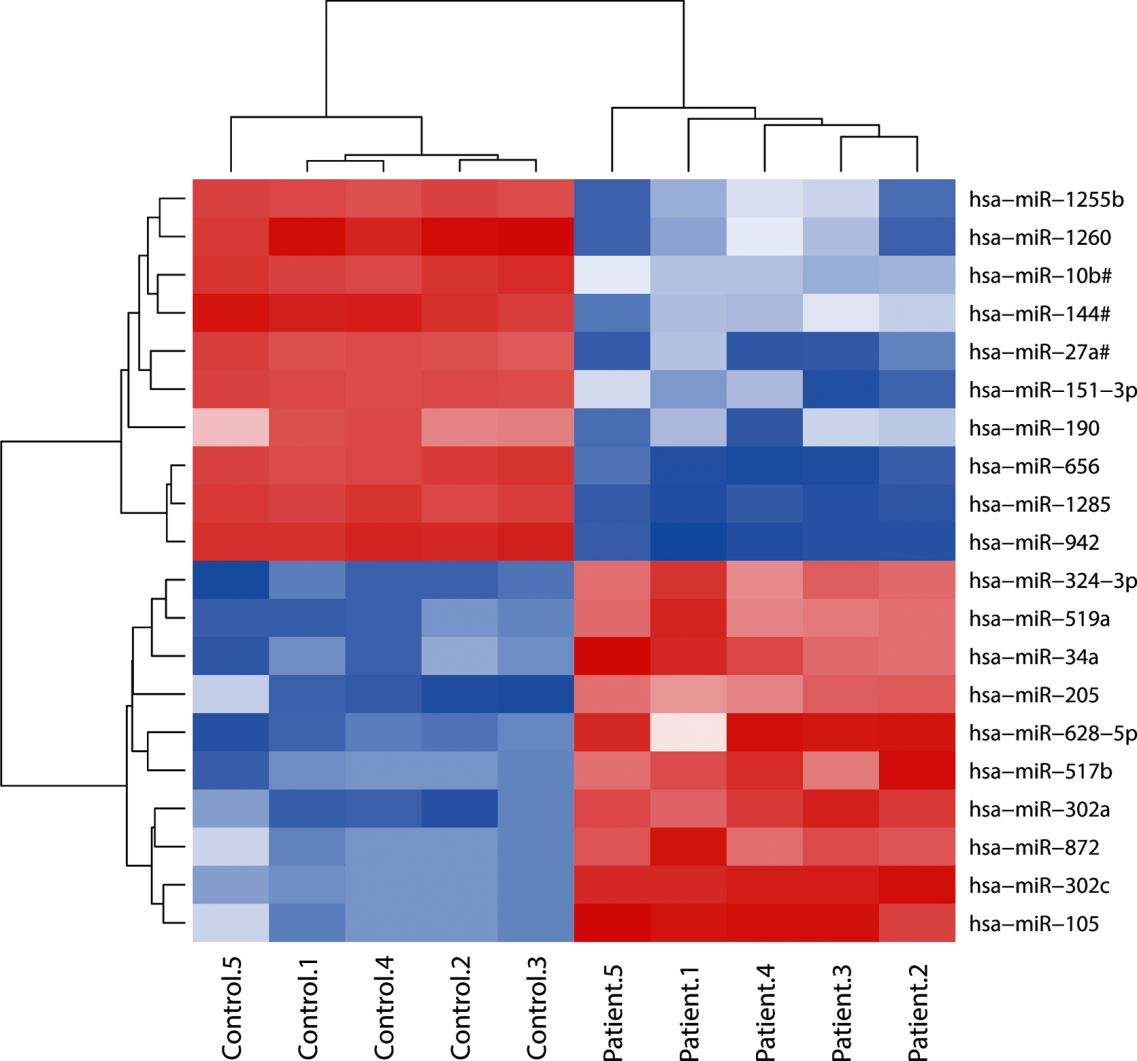
Heat-map of the two-way hierarchical clustering analysis of the 20 most dysregulated miRNAs in LSCC plasma samples vs healthy controls. The red and blue colors indicate expression level of miRNAs in terms of normalized Ct value.

TLDA was performed to profile differentially expressed miRNAs in plasma samples from stage I LSCC patients. After excluding low abundant miRNAs (Ct value > 37 and non-detectable in over 25% of samples), The 20 most significant dysregulated miRNAs (up-regulated: miR-105, miR-302a, miR-302c, miR-324-3p, miR-517b, miR-519a, miR-872, miR205, miR-628-5p, and miR-34a; down-regulated: miR-190, miR-10b#, miR-1255b, miR1260, miR1285, miR144#, miR151-3p, miR-27#, miR-942, and miR-656) were selected for further analysis. The heat-map and clustering analysis showed that these candidate miRNAs were able to separate cancer patients from healthy controls (Figure 2).

### Selection of candidate plasma miRNAs as biomarker for early stage LSCC

Next, we further assessed and validated these 20 miRNAs in the training and validation sets of plasma samples containing 30 cases of each stage I LSCC patients and healthy controls.

In the training set, our data showed the 13 miRNAs were differentially expressed between LSCC patients and healthy controls, but 9 of them were inconsistent with TLDA results (Table 2). Thus, the remaining four miRNAs (miR-324-3p, miR-628-5p, miR-1285, and miR-302c) were selected for further evaluation.

**Table 2:** Validation of 20 differentially expressed miRNAs in the training set

miRNA	Control (*n* = 30)		LSCC (*n* = 30)		*P*	Expression Pattern
	Mean ± SD	Median	Mean ± SD	Median		TLDA Set/Training Set
miR-144#	16.62 ± 0.80	16.68	15.87 ± 1.17	16.11	0.004	Down/up
miR-105	13.39 ± 1.94	13.34	13.79 ± 1.38	13.57	0.695	Up/Down
miR-27a#	17.41 ± 1.28	17.22	16.40 ± 1.56	16.39	0.010	Down/Up
miR-151-3p	12.05 ± 2.12	11.75	8.75 ± 1.50	8.80	< 0.0001	Down/Up
miR-190	17.39 ± 1.31	17.83	14.15 ± 1.48	14.28	< 0.0001	Down/Up
miR-205	16.74 ± 0.91	16.66	15.17 ± 6.15	16.48	0.313	Up/Up
**miR-302c**	**13.55 ± 0.58**	**13.44**	**12.11 ± 0.62**	**12.27**	**< 0.0001**	**Up/Up**
**miR-324-3p**	**11.83 ± 1.27**	**11.95**	**10.17 ± 0.96**	**9.92**	**0.008**	**Up/Up**
**miR-628-5p**	**11.47 ± 0.91**	**11.37**	**10.81 ± 0.62**	**10.79**	**0.001**	**Up/Up**
miR-656	7.06 ± 0.44	7.20	5.56 ± 0.77	5.68	< 0.0001	Down/Up
miR-872	8.40 ± 1.64	8.67	9.67 ± 0.83	9.71	< 0.0001	Up/Down
miR-942	17.69 ± 1.16	17.88	16.98 ± 1.40	17.25	0.057	Down/Down
**miR-1285**	**14.10 ± 1.60**	**13.63**	**18.04 ± 1.84**	**18.36**	**< 0.0001**	**Down/Down**
miR-1260	13.26 ± 0.91	13.06	11.73 ± 1.36	11.81	0.003	Down/Up
miR-1255b	16.78 ± 0.59	16.82	17.04 ± 0.57	17.03	0.057	Down/Down
miR-302a	Undetected		Undetected		NA	Up/-
miR-519a	18.59 ± 1.43	18.89	18.39 ± 1.37	17.82	0.110	Up/Up
miR-517b	Undetected		Undetected		NA	Up/-
miR-34a	15.07±1.35	15.29	15.97 ± 1.07	16.24	0.005	Up/Down
miR-10b#	18.03±1.44	18.36	17.80 ± 1.31	17.25	0.015	Down/Up

LSCC, lung squamous cell carcinoma.

**Figure 3:**
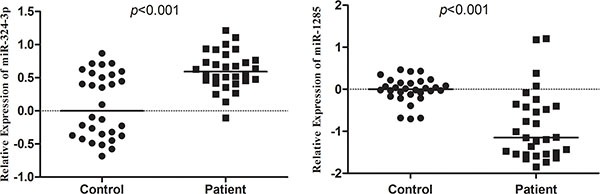
Plasma levels of miR-324-3p and miR-1285 expression in a validation set of samples Plasma expression levels of miR-324-3p and miR-1285 in validation set were analyzed using qRT-PCR and normalized with cel-miR-39. Expression data are represented as log_10_^2−ΔΔCt^ values.

In the validation set, we validated plasma levels of these four miRNAs using qRT-PCR. Compared to healthy controls, miR-324-3p was significantly up-regulated, whereas miR-1285 was significantly down-regulated in stage I LSCC patients (Figure 3). However, miR-628-5p and miR-302c did not show any significant difference in the training cohort. Since only plasma miR-324-3p and miR-1285 showed consistence expression pattern among TLDA set, training set and validation set, we focused on investigating these 2 miRNAs in the subsequent studies.

**Figure 4:**
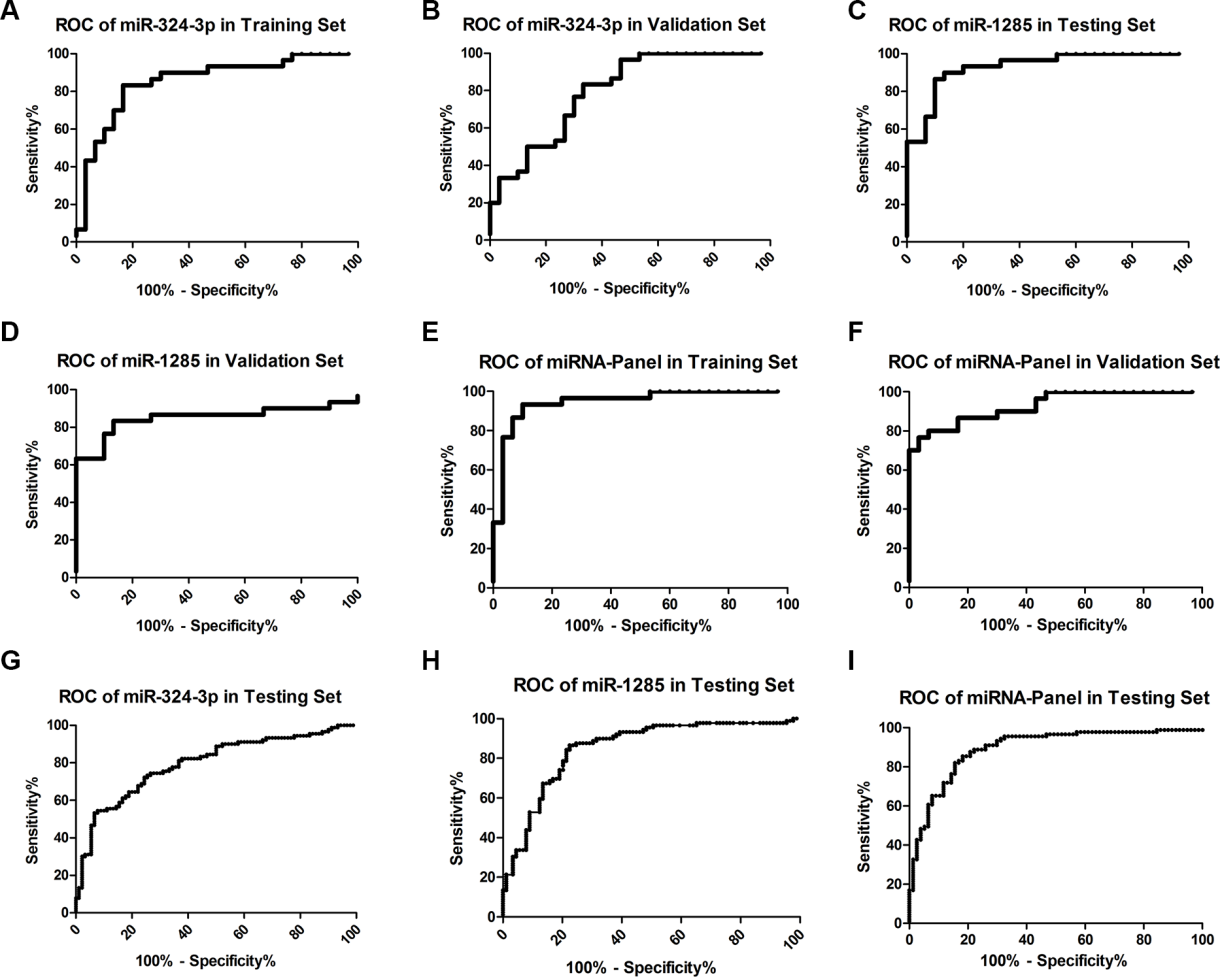
ROC curves of miRNAs in the different cohorts of samples ROC curves of miR-324-3p, miR-1285, and their combination were established for these three cohorts of samples. (**A**) miR-324-3p in the training Set; (**B**) miR-324-3p in the validation set; (**C**) miR-1285 in the training set; (**D**) miR-1285 in the validation set; (**E**) 2-miR-panel in the training set (**F**) 2-miR-panel in the validation set. (**G**) miR-324-3p in the testing set; (**H**) miR-1285 in the testing set; (**I**) 2-miR-panel in the testing set.

We then further investigated the potential of two plasma miRNAs as diagnostic biomarkers for early stage LSCC by generating the receiver operating characteristic (ROC) curves (Figure 4). Our data showed that the area under the curve (AUC) of miR-324-3p in the training set (Figure 4A) and validation set (Figure 4B) was 0.85 (95% confidence interval (CI) = 0.75 to 0.95; sensitivity = 83.3%, specificity = 83.3%) and 0.80 (95% CI = 0.69 to 0.91; sensitivity = 83.3%, specificity = 66.7%), respectively, while the AUC of miR-1285 in the training set (Figure 4C) and validation set (Figure 4D) was 0.93 (95% CI = 0.87 to 0.99; sensitivity = 90.0%, specificity = 86.7%) and 0.85 (95% CI= 0.74 to 0.96; sensitivity = 83.3%, specificity = 86.7%), respectively.

To evaluate the joint performances of these two miRNAs in discriminating the stage I LSCC patients from healthy controls, a combination of the expression levels of the miRNAs weighted by the regression coefficient was used to construct a risk score in the training cohort. The AUC of the miRNA panel was 0.95 (95% CI = 0.89 to 1.00; sensitivity = 93.3%, specificity= 90.0%) for the training set (Figure 4E) and 0.93 (95% CI= 0.87 to 0.99; sensitivity = 76.7%, specificity = 96.7%) for the validation set (Figure 4F), indicating that the combination of these two-miRNAs has a higher diagnostic value than single one.

### Validation of plasma miR-324-3p and miR-1285 in early LSCC detection

We then assessed these two miRNAs in the testing set of 90 cases of each stage I LSCC and healthy controls. The AUC of miR-324-3p (Figure 4G) and miR-1285 (Figure 4H) were 0.79 (95% CI = 0.73 to 0.86; sensitivity = 72.2%, specificity = 75.6%) and 0.85 (95% CI = 0.79 to 0.91; sensitivity = 86.5%, specificity = 77.5%), respectively. The AUC of the two-miRNA panel was 0.89 (95% CI = 0.84 to 0.93; sensitivity = 85.4%, specificity = 81.8%; Figure 4I).

**Figure 5:**
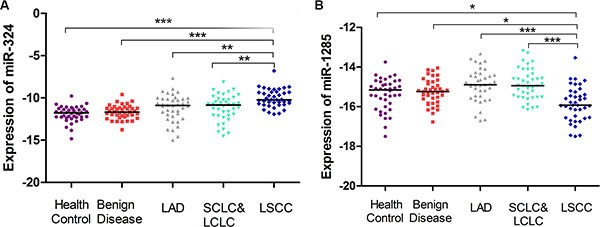
Differential expression of miR-324-3p and miR-1285 in the different histological types of lung cancer and benign diseases Plasma levels of miR-324-3p (**A**) and miR-1285 (**B**) in LSCC (blue), LAD (gray), SCLC/LCLC (green), benign disease (red) and healthy control (purple) were analyzed in the testing cohort of plasma samples using qRT-PCR. *p < 0.05; **p < 0.01, ***p < 0.001 using Kruskal–Wallis test.

Moreover, in order to explore whether these two miRNAs were specific in LSCC, we assessed their plasma levels in other histological types of lung cancer and lung benign disease. As shown in Figure 5, these two miRNAs was able to distinguish LSCC from LAD, large cell lung cancer (LCLC) and small cell lung cancer (SCLC). In addition, a significant difference in the expression levels of miRNA-324-3p and miR-1285 between LSCC and lung benign disease was observed. These results indicated that miR-324-3p and miR-1285 are indeed LSCC-specific.

### The expression level of plasma miR-324-3p and miR-1285 in other tumor types

**Figure 6:**
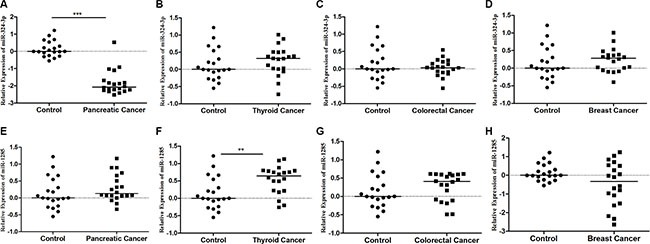
Expression of miR-324-3p and miR-1285 in the plasma of pancreatic cancer, thyroid cancer, colorectal cancer and breast cancer (**A**) miR-324-3p in pancreatic cancer; (**B**) miR-324-3p in thyroid cancer; (**C**) miR-324-3p in colorectal cancer; (**D**) miR-324-3p in breast cancer; (**E**) miR-1285 in pancreatic cancer; (**F**) miR-1285 in thyroid cancer. (**G**) miR-1285 in colorectal cancer (**H**) miR-1285 in breast cancer. Expression data are represented as log10^2−ΔΔCt^ values. **p < 0.01, ***p < 0.001.

Compared to healthy controls, plasma miR-324-3p expression levels were significantly decreased in pancreatic cancer (*p* < 0.0001), whereas plasma miR-1285 expression level was significantly increased in thyroid cancer (*p* < 0.001). However, in thyroid cancer, colorectal cancer and breast cancer, there were no significant differences of miR-324-3p compared with healthy controls. And no significant differences of plasma level of miR-1285 were observed in pancreatic cancer, thyroid cancer and colorectal cancer (Figure 6). The discrepancy of the miR-324-3p and miR-1285 between cancer and control in these tumor types were inconsistent with those in LSCC. Therefore, these results further supported the specificity of the changes of miR-324-3p and miR-1285 in early stage LSCC.

### Correlation between the plasma level of the 2 miRNAs and clinical parameters

We examined the association of plasma levels of miR-324-3p and miR-1285 with clinical factors in early-stage LSCC. No significant differences were observed when LSCC patients were stratified by gender and age (Supplementary Figure S1).

### Association of plasma miR-324-3p and miR-1285 levels with LSCC prognosis

**Figure 7:**
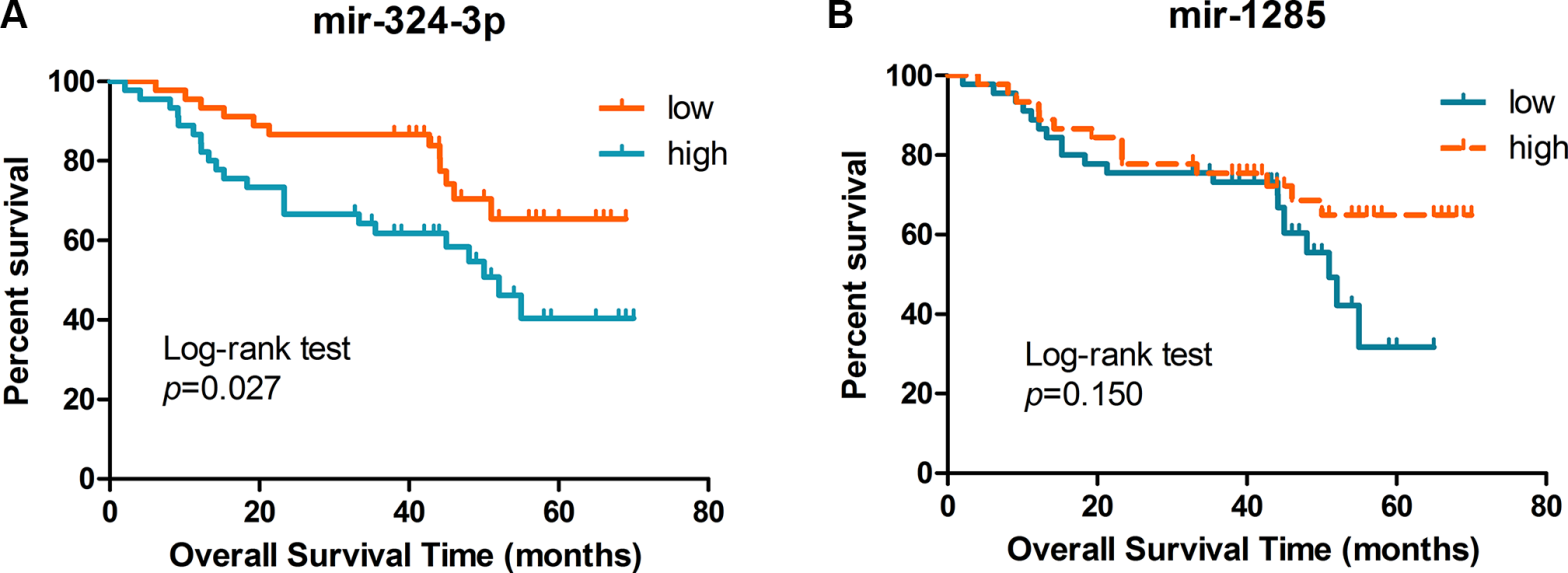
Kaplan-Meier curves of the overall survival stratified by plasma levels of miR-324-3p and miR-1285 in the testing set of samples Association of overall survival of patients with plasma level of miR-324-3p (**A**) and miR-1285 (**B**) was plotted and analyzed using the log-rank test.

To further determine whether plasma miR-324-3p and miR-1285 levels are predictive of prognosis, we performed an analysis of overall survival(OS) in all patients from the testing set. The 90 patients were divided into high and low plasma levels of miR-324-3p or miR- 1285 groups using a cut-off point of the median expression value. Kaplan-Meier analysis revealed that patients with high plasma levels of miR-324-3p had poor OS (*p* = 0.027, using the log-rank test), whereas plasma miR-1285 levels failed to predict OS of these patients (*p* = 0.15, using the log-rank test; Figure 7).

The univariate analysis demonstrated that high plasma miR-324-3p level was a significant prognostic indicator for OS of stage I LSCC patients (hazard ratio = 2.169, 95% CI: 1.072–4.388, *p* = 0.031). The multivariate analysis showed that plasma level of miR-324-3p was an independent prognostic predictor for stage I LSCC patients (hazard ratio = 3.039, 95% CI: 1.340–6.894, *p* = 0.008; Table 3).

**Table 3:** Univariate and multivariate analysis of overall survival of patients

Characteristic	Univariate analysis		Multivariate analysis	
	HR (95% CI)	*P* value	HR (95% CI)	*P* value
Sex (male vs. female)	1.190 (0.536–2.641)	0.669	0.658 (0.256–1.693)	0.385
Age (< 60 vs. ≥ 60 years)	1.071 (0.536–2.140)	0.846	1.004 (0.462–2.181)	0.993
Smoking status	1.984 (0.963–4.086)	0.063	2.627 (1.147–6.013)	**0.022**
SCC	0.757 (0.369–1.556)	0.449	0.698 (0.328–1.485)	0.351
Cyfra-21-1	0.847 (0.591–1.290)	0.497	0.773 (0.421–1.419)	0.406
miR-324-3p	2.169 (1.072–4.388)	**0.031**	3.039 (1.340–6.894)	**0.008**
miR-1285	0.606 (0.304–1.209)	0.155	0.572 (0.277–1.180)	0.130

### *In silico* analysis of target gene network and pathways

**Figure 8:**
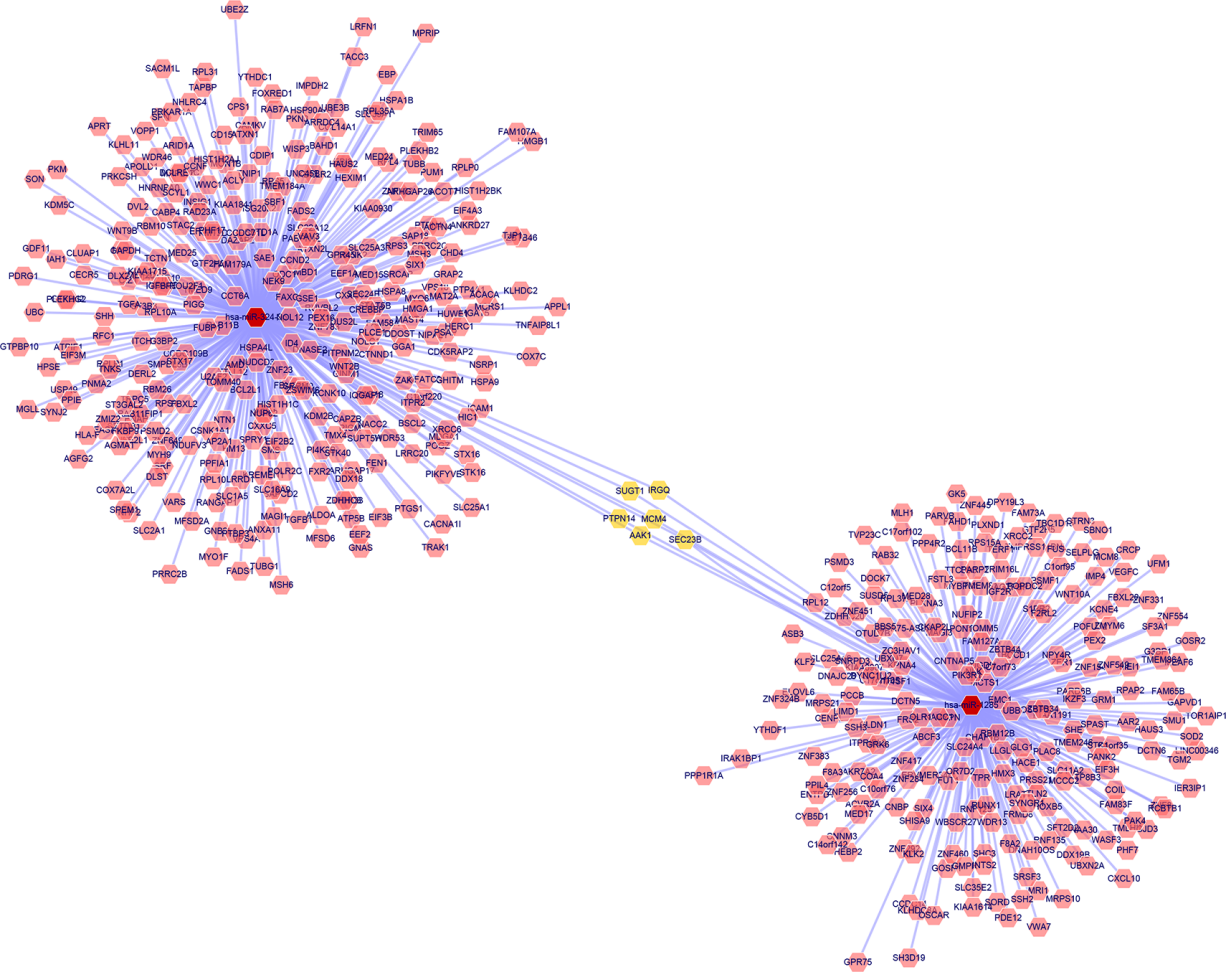
Bioinformatic analysis of miRNAs-regulated gene network Red nodes refer to miRNAs, pink nodes refer to the target genes, and yellow nodes refer to the targets shared by both miRNAs.

The list of the experimentally validated target genes of miR-324-3 and miR-1285 was downloaded from miRecords. By using Cytoscape, these two miRNA-target interactions were visualized as a network containing the 2 miRNAs and their target genes (Figure 8). Each interaction consisted of two nodes: a miRNA node (red) and a target gene node (pink). Overall, 560 target genes were identified, 331 of which were targeted by miR-324-3p and 235 by miR-1285. The network analysis showed that six targets (AAK1, IRGQ, SEC23B, MCM4, PTPN14, and SUGT1) were shared by both miRNAs.

Next, the validated miRNA-target interactions on pathways from KEGG and GO were obtained from miRWalk 2.0. The results showed that the miRNA target genes involved in many important pathways associated with cancer development, e.g., adherin junction, Wnt, Hedgehog, TGF-beta, VEGF, and p53 signaling pathways (Table 4). These results indicated a potentially critical functional role of miR-324-3p and miR-1285 in the progression of lung cancer.

**Table 4:** Results of pathway analysis of the target genes

Pathway	Target gene
Wnt signaling pathway	CCND2, CREBBP, CSNK1A1, DVL2, NFATC3, WNT9B
Hedgehog signaling pathway	WNT9B, CSNK1A1
Adherin junction	ACTN4, CREBBP, CTNND1, TJP1, IQGAP1
TGF beta signaling pathway	ID4, CREBBP
VEGF signaling pathway	NFATC3
Pathways in cancer	CREBBP, DVL2, MSH6, HSP90AA1, SLC2A1, STAT5B, APPL1
p53 signaling	TP53

## DISCUSSION

Accumulating evidence suggests that circulating miRNAs are of great value in diagnosis and prognosis of human cancer. miRNAs are packaged in extracellular vesicles such as exosome and released into the extracellular environment. In a paracrine manner, circulating miRNAs can be transferred into recipient cells to exert their biological functions [9]. These findings indicate that the circulating miRNAs are not only useful as biomarkers for tumor load but also contribute to tumor development and progression. Recently, Sozzi *et al.* reported that a plasma miRNA signature could effectively reduce the false-positive rate of LDCT, indicating the role of miRNAs in improving the efficacy of the traditional diagnostic tools [10]. Although several studies had identified a number of miRNAs, most of these studies did not accurately analyze the expression of miRNAs in the different histotypes and stages. Since the miRNA expression patterns are quite different in the distinct pathological types and tumor stages, the miRNAs identified in those studies may have less power in lung cancer screening for high-risk asymptomatic population.

In the current study, we used a multi-phase design and enrolled only stage I LSCC patients to identify plasma miRNA for early detection of LSCC. We first profiled differentially expressed plasma miRNAs between stage I LSCC patients and cancer-free controls using TLDA technology and identified the most significantly altered 20 miRNAs. However, our current study did not reveal some of the commonly reported dysregulated miRNAs in lung cancer, such as Let-7, miR-21, or miR-155 [11, 12]. One of the possible explanations for these missing miRNAs might be the different profiling tools used in these studies. Furthermore, this discrepancy might also result from the heterogeneity of the histological subtypes and tumor stages.

In the subsequent three-stage validation, our current data showed that miR-324-3p and miR-1285 could be highly promising as diagnostic biomarkers for early stage LSCC. Using a logistic regression model, the two-miRNA panel showed even higher sensitivity and specificity, which is much better than traditional blood markers, such as squamous cell carcinoma antigen and cyfra-21-1. Moreover, multivariate analysis revealed that over-expression of plasma miR-324-3p could predict a poor overall survival of early stage LSCC patients. Since miRNA expression profiles of LSCC dramatically differ from other histological subtypes of lung cancer, the expression level of miR-324-3p and miR-1285 were also evaluated in plasma samples of LAD, LCLC, SCLC, hamartoma and inflammatory pseudotumor. The results showed that altered levels of miR-324-3p and miR-1285 only occurred in the LSCC plasma. In addition, we also assessed the plasma expression of miR324-3p and mir-1285 in other tumor types including pancreatic cancer, thyroid cancer, colorectal cancer and breast cancer. The discrepancy of the miR-324-3p and miR-1285 between cancer and control in these tumor types were different from those in LSCC. Thus, these results supported the specificity of the expression pattern of miR-324-3p and miR-1285 in early stage LSCC.

To gain a further insight into the functional role of miR-324-3p and miR-1285, we retrieved the targeting genes of these two miRNAs and analyzed their related pathways. Bioinformatic analysis revealed that some important tumor-related genes are simultaneously regulated by these two miRNAs. For example, PTPN14 is a non-receptor tyrosine phosphatase and can interact with dephosphorylated β-catenin. The repression of PTPN14 could promote intrahepatic cholangiocarcinoma cell growth [13]. The overexpression of Sugt1, a cochaperone of Hsp90, has been found in many cancer types including LSCC and contributes to the development of these cancers [14]. Moreover, AAK1, adaptor-associated kinase 1 was shown to be a positive regulator of the NOTCH pathway, which is critical in carcinogenesis [15] Cao et al. reported that down-regulation of Notch pathway could induce apoptosis in lung squamous cell carcinoma cells [16]. Thus, miR-1285 and AAK1 interaction may involve in this process. In addition, we also found that these two miRNAs could regulate some crucial pathways. Dysregulation of TGF-beta signaling pathway is important in cancer progression and cell invasion. Pajares et al. found that TGF-beta-induced protein expression is an independent predictor of survival in adjuvant-treated lung squamous cell carcinoma patients. Our informatics analysis identified two target genes(ID4 and CREBBP) of miR-324-3p which involved in TGF-beta signaling pathway. Yang et al. found that high expression levels of VGEF-B is associated poor survival of LSCC patients. Several target genes related to VGEF signaling pathway have been identified. However, further molecular investigations are needed to confirm these predictions.

Dysregulated plasma mir-324-3p levels have been observed in several studies. For example, Yang et al. reported that miR-324-3p, together with miR-20a-5p and miR-320a, could serve as a diagnostic biomarker for the early detection of hepatocellular carcinoma (HCC) [17]. Namkung *et al.* showed that miR-324-3p was down-regulated in pancreatic cancer tissues and had the largest effect sizes of the 19-miRNA signature for predicting the outcome of pancreatic cancer [18]. The down-regulation of miR-324-3p, observed in nasopharyngeal carcinoma cell lines, contributed to the acquisition of cancer cell radioresistance [19]. In line with our study, Liu *et al.* reported that expression levels of miR-1285 are significantly downregulated in plasma of HCC and could serve as a biomarker for HCC patients received transarterial chemoembolization. Moreover, they showed that miR-1285-3p could directly repress expression of JUN oncogene in HCC cells, indicating a potential tumor suppressor role of miR-1285-3p [20]. However, the level of miR-1285 in bronchial lavage samples of lung cancer patients was up-regulated compared to those of patients with benign lung disease [21]. The controversial results again reveal and confirm the heterogeneity of miRNA expression patterns among cancer and sample types. In addition, the complexity of mechanism involved in the release of miRNAs from its parental cells may also contribute to the inconsistency of the results [9].

In summary, our current study identified plasma levels of miR-324-3p and miR-1285 as potential diagnostic biomarkers for early stage LSCC. Detection of plasma mir-324-3p levels may also serve as a prognostic marker for LSCC patients. Future studies using a larger sample size from multiple institutions are warranted to validate our current finding for clinical practice. The functional investigation is also required to explore the underlying mechanisms of these miRNAs in LSCC development.

## MATERIALS AND METHODS

### Study design and patients

This study was approved by the institutional review board of Tianjin Medical University Cancer Institute and Hospital (Tianjin, China) and all patients provided a written informed consent form before enrolled into the study. All experiments were performed in accordance with relevant ethnic guidelines and regulations.

In total, we enrolled 395 patients and 195 age and gender-matched healthy control individuals. The study was divided into three phases including screening, selection and testing phases. The patients were randomly assigned into these three phases. In the screening phase, 5 stage I LSCC patients were randomly selected. miRNA expression levels in plasma collected from stage I LSCC patients (*n* = 5) and healthy controls (*n* = 5) were profiled by using TaqMan Low Density Array (TLDA). In the selection phase, the candidate miRNAs selected in screening phase were tested in two independent cohorts (the training cohort, 30 stage I LSCC patients vs. 30 controls; validate cohort, 30 Stage I LSCC patients vs. 30 controls) using individual TaqMan probe-based quantitative reverse-transcription polymerase chain reaction (qRT-PCR) assays. In the testing phase, another independent cohort (stage I LSCC patients *n* = 90 and healthy controls, *n* = 90) was used to validate the potential miRNA candidates for the early detection of LSCC. Furthermore, in order to assess whether the candidate miRNAs are specific for LSCC, a comparison set of plasma samples was used. This set of samples included patients with stage I LSCC (*n* = 40), stage I LAD (*n* = 40), large cell lung cancer (LCLC, *n* = 17), small cell lung cancer (SCLC, *n* = 23) and benign lesions (hamartoma, *n* = 25; inflammatory pseudotumor, *n* = 15) as well as healthy controls (*n* = 40). To determine the expression level of the candidate miRNAs in other tumor types, the plasma samples of pancreatic cancer (*n* = 20), thyroid cancer (*n* = 20), colorectal cancer (*n* = 20) and breast cancer (*n* = 20) were also collected. Finally, the prognostic value of the candidate miRNAs was evaluated in the testing set. The overview of the study is illustrated in Figure 1.

These patients underwent medical treatment in our Cancer Institute and Hospital between June 2009 and August 2015 and healthy controls received health check in the Cancer Institute and Hospital at the same period of time. The stage of the tumor was assessed according to the UICC/TNM classification. All patients didn’t receive any chemoradiation therapy before surgery and blood withdrawal. Each participant provided 5 mL venous blood sample for this study.

LSCC patients in the testing set of samples were followed up regularly through telephone interview and the last follow-up was conducted in December 2015.

### Preparation of plasma and RNA isolation

The blood (5 ml) was collected in an ethylenediaminetetra acetic acid-anticoagulant vacuum tube and processed within 2 h. To prepare plasma sample, plasma was separated from blood samples by centrifugation at 1,300 rpm for 20 min at room temperature and the plasma was carefully collected and transferred into a 1.5 ml RNAase-free tube and stored at −80°C until use.

Total RNA was isolated from 400 μL plasma using a mirVana miRNA Isolation Kit (Ambion, Austin, TX, USA) according to the manufacturer's protocol. To allow for normalization of sample-to-sample variations of the RNA isolation efficiency, synthetic *C. elegans* miRNA (cel-miR-39) was added to each sample according to a previous study [22]. The RNA samples were preserved at −80°C until use. Repeated freeze-thawing was avoided to ensure the quality of the samples during storage.

### TLDA and qRT-PCR

Total RNA was subjected to reverse transcription (RT) using the TaqMan miRNA RT kit and megaplex RT primers (Applied Biosystems) following the manufacturer's protocols. To increase the detection sensitivity of the TLDA, a pre-amplification step was performed after the RT. MiRNA profiling of 757 different human miRNAs was then performed using the TLDA with an ABI PRISM 7900HT Sequence Detection System (TaqMan Array Human MicroRNA A + B Cards Set v3.0, Applied Biosystems). Data were analyzed with SDS Relative Quantification Software version 3.0.1 (Applied Biosystems).

In further validation phases, a TaqMan probe–based qRT-PCR assay was performed for quantitative determination of plasma miRNAs according to the manufacturer's instructions (7500 Sequence Detection System, Applied Biosystems) as described previously [23]. All reactions were conducted in triplicate. The expression levels of the miRNAs were presented as threshold cycle (Ct) values and normalized to cel-miR-39. Relative content was calculated using the comparative Ct method (2^−ΔΔCt^).

### Bioinformatic analysis of miRNA-target gene network and pathway

The list of validated target genes of the candidate miRNAs were obtained from miRecords v4.0 (http://www.mirecords.biolead.org) database, which offers a comprehensive data of possible miRNA-targets of 11 different data sets. The validated miRNA-target interactions on pathways from Kyoto Encyclopedia of Genes and Genomes (KEGG) categories and Gene Ontology (GO) categories were downloaded from miRWalk 2.0 [24]. Biological networks were created using Cytoscape v3.2 open-source software.

### Statistical analysis

The TLDA data were analyzed using *t*-test and Benjamini Hochberg correction for false discovery rate such that differential expression was considered to be significant with a *p*-value < 0.01. The validation of plasma qPCR expression data was analyzed using nonparametric Mann–Whitney *U*-test for two groups and Kruskal-Wallis test for multiple groups. The difference (association) in demographic and clinicopathological characteristics between case and control was analyzed using the χ^2^ test or Fisher's exact test for qualitative data and Student's *t*-test for quantitative data. The receiver operating characteristic (ROC) curves were established to determine the sensitivity, specificity, and area under the curve (AUC) for plasma miRNAs. The overall survival (OS) was calculated from the date of a definitive diagnosis to death or to the date of the last follow up. The OS curves were evaluated by the Kaplan–Meier method and analyzed using the log-rank test. The Cox proportional hazard regression analysis was used to identify candidate miRNAs with independent prognostic values. All statistical analyses were performed using SPSS statistical package, version 20.0 for Windows (SPSS Inc., Chicago, IL, USA). All tests were two tailed and a *p*-value ≤ 0.05 was considered statistically significant. Graphical plots were generated using GraphPad Prism version 5.00 for Windows (GraphPad Software, San Diego, CA, USA).

## SUPPLEMENTARY MATERIALS FIGURE


