# Supplementary material for: Plasma miR-324-3p and miR-1285 as diagnostic and prognostic biomarkers for early stage lung squamous cell carcinoma
Source: Oncotarget. 2016 Aug 11;7(37):59664–75. doi: 10.18632/oncotarget.11198 (PMC5312339; doi:10.18632/oncotarget.11198)
Supplement: Supplementary file 1 [file oncotarget-07-59664-s001.pdf]

Plasma miR-324-3p and miR-1285 as diagnostic and prognostic biomarkers for early stage lung squamous cell carcinoma

Supplementary Materials

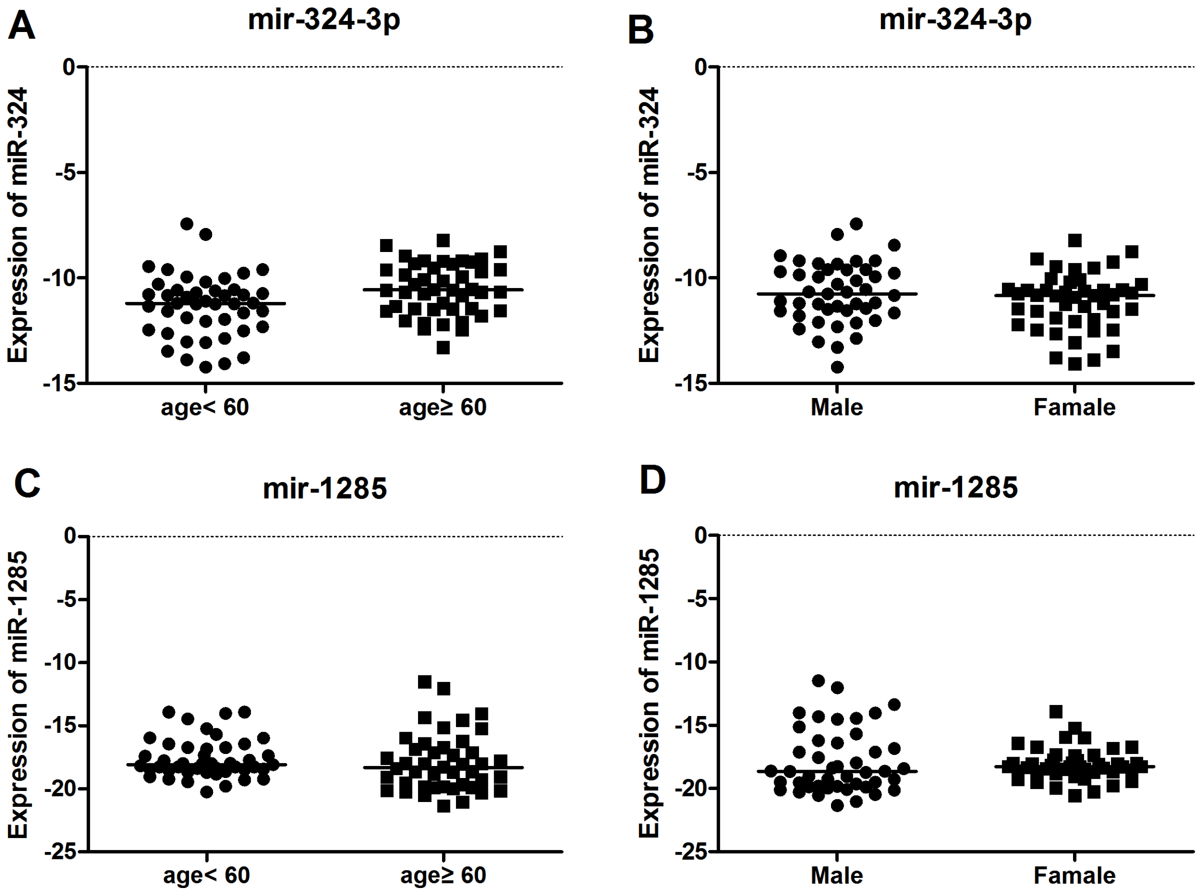

Supplementary Figure S1: The relationship of plasma miRNAs and clinical parameters. LSCC patients were stratified by gender and age. (**A**) plasma miR-324-3p expression between two age groups; (**B**) plasma miR-324-3p expression between male and female; (**C**) plasma miR-1285 expression between two age groups; (**D**) plasma miR-1285 expression between male and female.
